# Prognostic value of coronary computed tomography angiography compared to radionuclide myocardial perfusion imaging in patients With coronary stents

**DOI:** 10.3389/fcvm.2023.1087113

**Published:** 2023-03-17

**Authors:** Rami M. Abazid, Jonathan G. Romsa, James C. Warrington, Cigdem Akincioglu, Osama A. Smettei, Yves Bureau, Nikolaos Tzemos, William C. Vezina

**Affiliations:** ^1^ Division of Nuclear Medicine, London Health Sciences Centre, Victoria Hospital, London, ON, Canada; ^2^ Medical Biophysics, Western University, London, ON, Canada; ^3^ Department of Psycholoy, Lawson Health Research Institute, London, ON, Canada; ^4^ Division of Cardiology, Department of Internal Medicine, London Health Sciences Centre, University Hospital, London, ON, Canada

**Keywords:** stent, percutaneous coronary intervention, prognostic value, computed tomography angiography, single-photon emission computed tomography

## Abstract

**Objectives:**

The aim of this study is to compare the prognostic value of coronary computed tomography angiography (CCTA) with single-photon emission computed tomography (SPECT) in predicting cardiovascular events in patients with stents.

**Design:**

Retrospective analysis.

**Setting:**

University Hospital, London, Ontario Canada**.**

**Participants:**

Between January 2007 and December 2018, 119 patients post-percutaneous coronary intervention (PCI) who were referred for hybrid imaging with CTA and 2-day rest/stress SPECT were enrolled.

**Primary and secondary outcome measures:**

Patients were followed for any major adverse cardiovascular event (MACE) including: All-cause mortality, Non-fatal myocardial infarction (MI), Unplanned revascularization, Cerebrovascular accident and hospitalization for arrhythmia or heart failure. We define hard cardiac events (HCE) as: cardiac death, non-fatal MI or unplanned revascularization. We used two cut-off values to define obstructive lesions with CCTA ≥50% and ≥70% in any coronary segment. SPECT scan defined as abnormal in the presence of >5% reversible myocardial perfusion defect.

**Results:**

During the follow-up period of 7.2 ± 3.4 years. 45/119 (37.8%) patients experienced 57 MACE: Ten deaths (2 cardiac deaths and 8 of non-cardiac deaths), 29 acute coronary syndrome including non-fatal MI (25 required revascularization), 7 hospitalizations for heart failure, 6 cerebrovascular accidents and 5 new atrial fibrillation. 31 HCEs were reported. Cox regression analysis showed that obstructive coronary stenosis (≥50% and ≥70%) and abnormal SPECT were associated of MACE (*p =* 0.037, 0.018 and 0.026), respectively. In contrast, HCEs were significantly associated with obstructive coronary stenosis of ≥50% and ≥70% with *p* = 0.004 and *p* = 0.007, respectively. In contrast, abnormal SPECT was a nonsignificant predictor of HCEs *(p* = 0.062).

**Conclusion:**

Obstructive coronary artery stenosis on CCTA can predict MACE and HCE. However, abnormal SPECT can only predict MACE but not HCE in patients post-PCI with a follow-up period of approximately 7 years.

## Introduction

Percutaneous coronary intervention (PCI) is widely used for coronary artery revascularization through stent deployment in the coronary segments with significant stenosis (1). In-stent restenosis and thrombosis need repeat PCI in approximately 10% of patients following stent implantation (2). Invasive coronary angiography is considered the imaging modality of choice to assess coronary stents. Cardiac computed tomography angiography (CCTA) has a limited role in coronary stent evaluation due to partial volume effect and blooming artifacts from stent struts. Thus, CCTA is not routinely recommended in patients with small stent diameters of ≤3 mm (3).

Few studies have evaluated the diagnostic accuracy of CCTA in stents. Using third-generation dual-source CT patient-based diagnostic accuracy in detecting stent stenosis of ≥50% has been reported at 95.7%; however, the diagnostic accuracy was significantly less in those stents with a diameter <3 mm (88.9%) than in those with a larger stent calibre of ≥3 mm (98.4%) (4).

Stress/rest single-photon emission computed tomography (SPECT) myocardial perfusion imaging (MPI) is used to assess myocardial ischemia by comparing differences in myocardial perfusion at stress and rest. Ischemia is defined as a reversible perfusion defect. Reversible perfusion defects usually represent hemodynamically significant coronary artery stenosis or in-stent restenosis post-PCI (5). However, SPECT MPI has several potential disadvantages; it generally provides relative rather than absolute functional assessment and rarely misses balanced triple vessel coronary artery disease or left main disease despite the increased morbidity and mortality (6). SPECT MPI also has lower spatial resolution and does not provide precise anatomical information.

Hybrid imaging approaches are increasingly being used in the context of coronary artery disease. These approaches encompass anatomical imaging with CCTA or invasive coronary angiography combined with functional assessment with nuclear perfusion imaging, cardiac magnetic resonance or CT myocardial perfusion imaging. This results in increased diagnostic accuracy for the detection of coronary artery disease (CAD) and prediction of major adverse cardiovascular events (MACE) in patients with myocardial ischemia (7–9).

Data is lacking about hybrid imaging in patients with stents. In our study we compare the prognostic value of CCTA vs. SPECT MPI in predicting MACE in patients with stents.

## Methods

### Patient and public involvement

**Figure 1:**
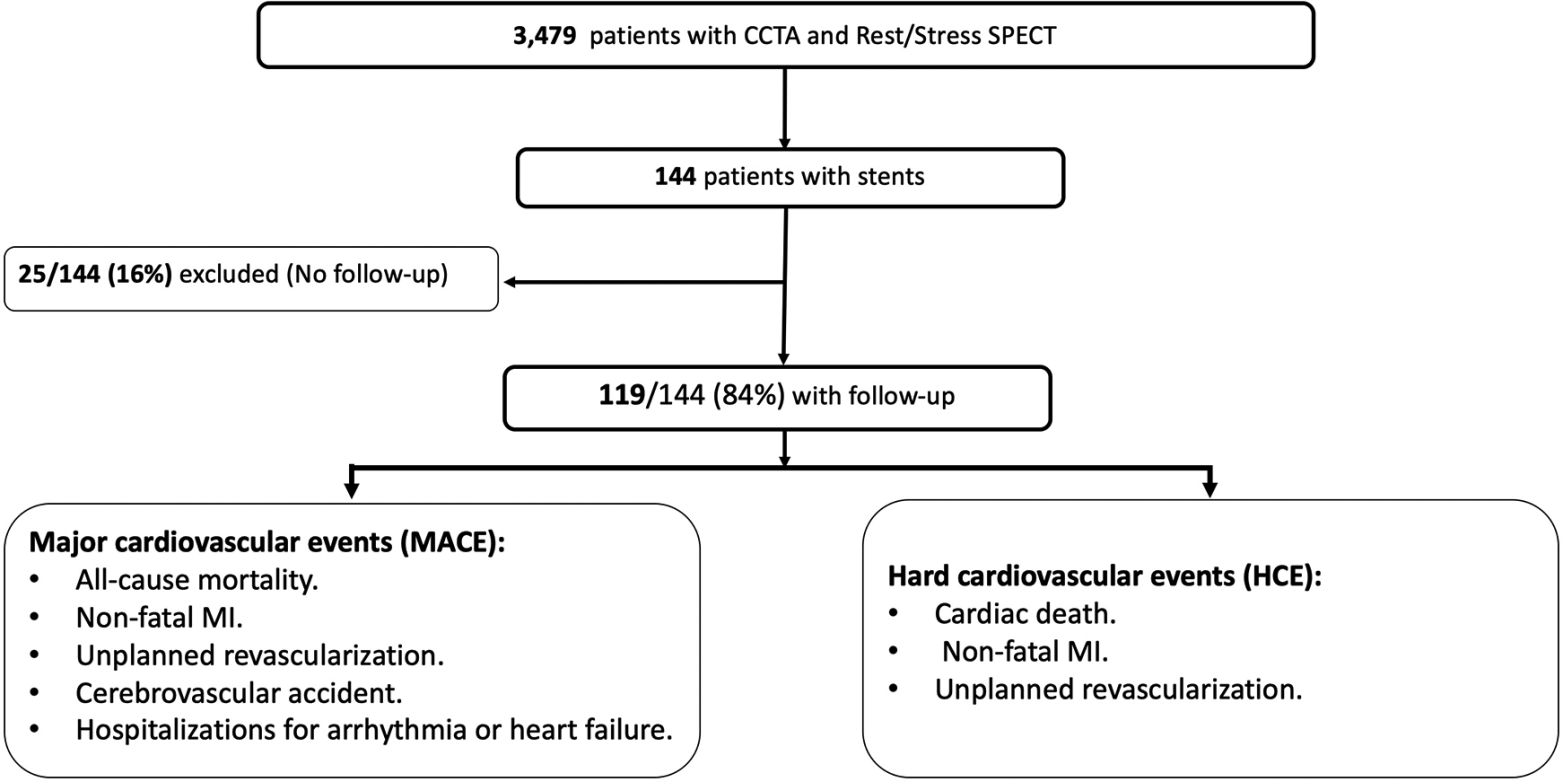
Patient inclusion.

Between January 2007 and December 2018, 144 consecutive patients post-PCI with chronic symptoms were referred for hybrid imaging with CCTA and 2-day rest/stress SPECT MPI and were retrospectively analyzed Figure 1. The exclusion criteria included: Patients who lost their follow up, patients who had only CCTA with no SPECT MPI imaging and patients who had PCI after coronary artery bypass surgery (CABG). However, patients who had PCI as a part of hybrid revascularization during CABG were included.

All patients signed an informed consent to be enrolled in a hybrid imaging registry (CCTA and SPECT MPI) with long-term follow-up. This study was approved by the local ethics committee.

### CCTA scan acquisition

A 64-slice CT scan was used for all patients (GE Healthcare, LightSpeed VCT, Chicago, IL, USA). All patients with heart rate >60 beats/minute received intravenous or oral beta blocker before the scan to lower heart rate. Sublingual 400 ug nitroglycerin was given before the scans if no contraindications. The scan protocol included a non-enhanced CT scan for calcium score measurement followed by the CCTA acquisition using a timing bolus technique to determine the contrast delay time.

### CCTA images interpretation

CCTA images were reconstructed and analyzed on GE Healthcare Advantage Workstations (version 4.4). For stent image processing, a sharp kernel was employed for edge enhancement. Agatston score was measured in all the coronary arteries excluding the stent locations. For the purpose of the study, two cut-off values were used to define obstructive lesions (≥50% and ≥70% stenosis) in coronary segments greater than 1.5 mm in diameter.

### SPECT MPI

A 2-day rest/stress protocol with Tc-99 m Sestamibi was used in all patients. The rest scans were performed on the same day as the CCTA. Stress scans were done the following day. Treadmill exercise was used for physically active patients while pharmacological stress with dipyridamole was used for those who were unlikely to perform adequately on the treadmill. Vasodilator stress was supplemented with supine cycle ergometer exercise if patient capable. A CZT gamma camera (GE Discovery NM 530c) was used for image acquisition in patients acquired from 2014 to 2018. A two headed sodium iodide gamma camera (GE Hawkeye) was used for patients acquired from 2007 to 2013. SPECT images were analyzed using cardiac software (Cedars QGS/QPS) and/or Emory Cardiac Toolbox (ECT) on GE Xeleris Nuclear Medicine workstations. Scans were defined as normal/near normal if perfusion defects were <5% of LV myocardial mass. Abnormal perfusion defects were defined as mild if involving 5%–10%, moderate if 10%–20% and large if >20% of the LV mass.

### Endpoints

Patients were followed for any major adverse cardiovascular event (MACE) including: (1) All-cause mortality, (2) Non-fatal myocardial infarction (MI), (3) Unplanned revascularization with PCI or CABG (unplanned if the procedure was performed more than three months after the indexed CTA), (4) Cerebrovascular accident, (5) Hospitalization for arrhythmia or heart failure. We defined hard cardiac events (HCE) as: (1) cardiac death, (2) non-fatal MI or (3) unplanned revascularization.

### Statistical analysis

Continuous variables are presented as means and standard deviations while categorical variables are presented as frequencies and percentages. We did descriptive statistics analysis using Wilcoxon rank-sum test to compare continuous variables and Pearson's chi-square test to compare categorical variables between patients with and without MACE. We also used univariable regression models to analyze the association of each independent clinical variables, CCTA measurements and SPECT MPI finding with outcome of MACE and HCE. Finally, we ran Kaplan Meier analyses using hazard-models to analyze the prognostic value of SPECT MPI and CCTA.

## Results

### Baseline characteristics

In total, 144 were included in the study. 25 patients were lost to follow-up and were excluded. 119/144 (84%) were included in the final analysis, Figure 1. The mean age at enrollment was 62 ± 9 years. Men were 87 (73.1%), other baseline characteristics are shown in Table 1.

**Table 1:** Baseline characteristics.

Variables	All patients
Number of patients (%)	119
Men (%)	87 (73.1)
Age (years), mean ± SD	62 ± 9
Diabetes Mellitus, *n* (%)	30 (25.2)
Hypertension, *n* (%)	87 (73.1)
Dyslipidemia, *n* (%)	90 (75.6)
Family history of coronary artery disease, *n* (%)	21 (33.6)
Smoker/remote smoker history, *n* (%)	39 (32.8)
Previous myocardial infarction, *n* (%)	39 (32.8)
LV Ejection fraction (%) mean ± SD	55 ± 8
Body mass index (kg/m^2^), mean ± SD	29.5 ± 6
Symptoms, *n* (%)	
Chest pain	71 (59.6)
Dyspnea	19 (15.9)
Others	29 (24.5)
Calcium score, mean ± SD	750 ± 769
Coronary artery bypass graft surgery, *n* (%)	49 (41.5)
Stent number, *n* (%)	
1 stent	75 (63)
2 stents	36 (30.3)
≥ 3stents	8 (6.7)
Coronary artery bypass graft surgery, *n* (%)	28 (23.)
CCTA ≥70%, stenosis, *n* (%)	32 (26.9)
CCTA ≥ 50% stenosis, *n* (%)	51 (42.9)
Revisable defects with SPECT, *n* (%)	38 (31.9)
Mild reversible perfusion defects	17 (14.3)
Moderate reversible perfusion defects	12 (10)
Severe reversible perfusion defects	9 (7.6)
History of atrial fibrillation	5 (4.2)
History of peripheral vascular disease	2 (1.7)
History of carotid stenosis	1 (0.8)
Chronic obstructive pulmonary disease	4 (3.4)

### Stents analysis

Majority of patients 75/119 (63%) had one stent. 36/119 (30.3%) had two stents. 8/119 (6.7%) had three stents or more. 46/175 (26.3%) of the stents had a diameter less than 3 mm. 76 stent were deployed in left anterior descending artery (LAD), 52 stents in the right coronary artery (RCA), 42 stents in left circumflex artery (LCx) and 5 stents in the left main coronary artery. 81/119 (68%) of the patients had either patent stents or non-critical stenosis, while 14/119 (11.8%) had significant in-stent restenosis >50% (8 patients with >70% / total occlusion). In 24/199 (20.2%) the stent was non-interpretable.

### SPECT analysis

In total, 67 perfusion defects were reported (38 reversible and 29 fixed). 17/38 (44.7%) of the *reversible* defects were in the stented coronary territories and 21/38 (55.3%) in non-stented coronary territories. 7 (24%) of the *fixed* perfusion defects were in the stented coronary artery territories while 22/38 (76%) were in the non-stented territories.

### Major and hard cardiac events

Endpoints (events) were during a follow-up period of 7.2 ± 3.4 years. In total, 45/119 (37.8%) patients experienced 57 MACE: Ten deaths (2 cardiac deaths and 8 non-cardiac deaths), 29 acute coronary syndrome including non-fatal MI (19 required PCI and 6 required CABG), 7 hospitalizations for heart failure, 6 cerebrovascular accidents, 5 new atrial fibrillation.

HCE were 31 (2 cardiac deaths and 29 of nonfatal MI and/or acute contrary syndrome requiring revascularization). Coronary artery disease risk factors and stent number did not differ between patients with and without MAEC or HCEs; other baseline characteristics of patients with and without MACE/HCE are illustrated in (Tables 2, 3).

**Table 2:** Univariate cox regression, comparison between patients with and without major adverse cardiovascular event (MACE).

Variables	MACE	No MACE	Cox Regression	*P* value
			HR (95% CI)				
Number of patients (%)	45 (37.8)	74 (62.2)		-
Men (%)	35 (77.8)	52 (70.3)	0.73 (0.36–1.48)	0.39
Age (years), mean ± SD	63 ± 10	62 ± 9	1.03 (0.99–1.06)	0.11
Diabetes Mellitus, *n* (%)	15 (33.3)	15 (20.3)	0.85 (0.44–1.65)	0.63
Hypertension, *n* (%)	35 (77.8)	52 (70.3)	0.72 (0.35–1.45)	0.36
Dyslipidemia, *n* (%)	33 (73.3)	57 (77)	0.74 (0.38–1.45)	0.38
Family history of CAD, *n* (%)	6 (13.3)	15 (20.3)	1.87 (0.79–4.44)	0.15
Smoking/remote smoking, *n* (%)	13 (28.9)	26 (35.1)	1.41 (0.74–2.69)	0.29
Previous myocardial infarction, *n* (%)	10 (32.3)	29 (33)	1.16 (0.62–2.17)	0.63
Ejection fraction (%) mean ± SD	55 ± 8	55 ± 7	1.01 (0.971.05)	0.48
Body mass index (kg/m^2^), mean ± SD	30.4 ± 7.3	29 ± 6	1.01 (0.97–1.05)	0.52
Symptoms, *n* (%)				
Chest pain	29 (64.4)	42 (56.8)	0.82 (0.41–1.62)	0.73
Dyspnea	7 (15.6)	12 (16.2)	1.09 (0.45–2.65)	0.57
Others	9 (20)	20 (27)	0.88 (0.43–1.33)	0.84
Calcium score, mean ± SD	816 ± 812	704 ± 740	1.02 (0.97–1.06)	0.13
Coronary artery bypass graft surgery, *n* (%)	11 (24.4)	17 (22.9)	1.10 (0.61–2.01)	0.73
Stent number, *n* (%)				
1 stent	27 (60)	48 (64.8)	9.1 (0.82–19.1)	0.91
2 stents	19 (42.2)	17 (22.9)	3.6 (0.61–14.2)	0.24
≥3 stents	4 (8.8)	4 (5.4)	0.77 (0.13–4.5)	0.87
CT ≥ 50% stenosis, *n* (%)	27 (60)	24 (32.4)	1.88 (1.04–3.45)	0.037
CT ≥70%, stenosis, *n* (%)	19 (42.2)	13 (17.6)	2.04 (1.14–3.70)	0.018
Reversible defects with SPECT, *n* (%)	20 (44.4)	18 (24.3)	1.96 (1.08–3.57)	0.026

**Table 3:** Univariable cox regression, comparison between patients with and without hard cardiac events (HCE).

Variables	HCE	No HCE	Cox Regression	*P* value
			HR (95% CI)				
Number of patients (%)	31 (26.1)	88 (73.9)		-
Men (%)	26 (83.9)	61 (69.3)	0.50 (0.19–1.31)	0.16
Age (years), mean ± SD	63 ± 11	62 ± 9	1.02 (0.93–1.06)	0.26
Diabetes Mellitus, *n* (%)	11 (35.5)	19 (21.6)	0.94 (0.42–2.10)	0.87
Hypertension, *n* (%)	23 (74.2)	64 (72.7)	0.88 (0.39–1.97)	0.75
Dyslipidemia, *n* (%)	22 (71)	68 (77.3)	1.16 (0.56–2.52)	0.70
Family history of coronary artery disease, *n* (%)	18 (72)	61 (66.3)	0.39 (0.12–1.29)	0.12
Smoking/remote smoking, *n* (%)	3 (9.7)	18 (20.5)	1.58 (0.71–3.54)	0.26
Previous myocardial infarction, *n* (%)	10 (32.3)	29 (33)	1.24 (0.58–2.64)	0.58
Ejection fraction (%) mean ± SD	56 ± 7	55 ± 8	1.03 (0.98–1.08)	0.25
Body mass index (kg/m^2^), mean ± SD	30.6 ± 5	29.1 ± 6	1.01 (0.96–1.07)	0.54
Symptoms, *n* (%)				
Chest pain	29 (64.4)	42 (56.8)	0.89 (0.56–1.31)	0.48
Dyspnea	7 (15.6)	12 (16.2)	0.31 (0.12–2.21)	0.65
Others	9 (20)	20 (27)	0.75 (0.43–1.23)	0.43
Calcium score, mean ± SD	871 ± 839	704 ± 741	1.01 (0.98–1.04)	0.13
Coronary artery bypass graft surgery, *n* (%)	9 (29)	19 (21.6)	0.60 (0.29–1.22)	0.16
Stent number, *n* (%)				
1 stent	22 (70.1)	53 (60.2)	11.7 (0.23–27.2)	0.94
2 stents	17 (37.8)	19 (25.7)	6.4 (0.71–19.5)	0.92
≥3 stents	3 (9.6)	5 (5.7)	0.43 (0.02–5.3)	0.91
CT ≥ 50% stenosis, *n* (%)	21 (67.7)	30 (34.1)	2.5 (1.23–5.26)	0.004
CT ≥70%, stenosis, *n* (%)	15 (48.4)	17 (19.3)	2.63 (1.22–5.55)	0.007
Reversible defects with SPECT, *n* (%)	14 (45.2)	24 (27.3)	1.92 (0.39–3.85)	0.062

### Prediction of MACE and HCE

Univariate Cox regression analysis showed that coronary artery stenosis of ≥50% and ≥70% and abnormal MPI were significant predictors of MACE (*p* = 0.037, 0.018 and 0.026), respectively, Table 2 In contrast, HCEs were significantly predicted with coronary stenosis of ≥50% and ≥70% (*p *= 0.004, 0.007), respectively; while abnormal SPECT did not predict HCEs (*p* = 0.062,) Table 3. Multivariable regression models including all variables that were significant with the univariable regression analysis and found that none of these variables have a significant prediction of MACE or HACE.

### Survival analysis

**Figure 2:**
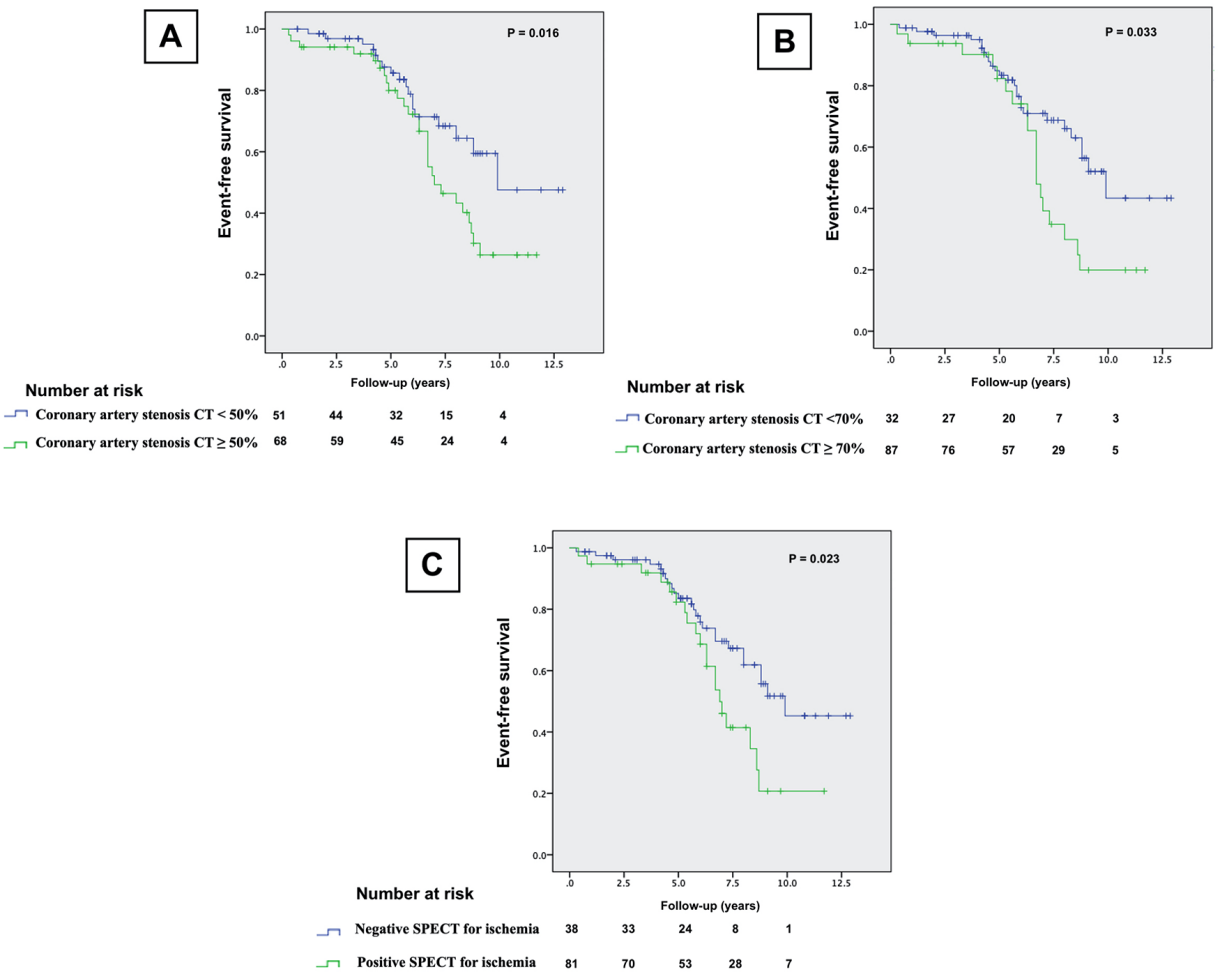
Kaplan-Meier MACE-free survival curves, (**A**): CCTA coronary stenosis ≥50% stenosis compared to <50% stenosis, (**B**): CCTA coronary stenosis of greater than 70% versus <70%, and (**C**): patients with and without revisable ischemia on SPECT.

**Figure 3:**
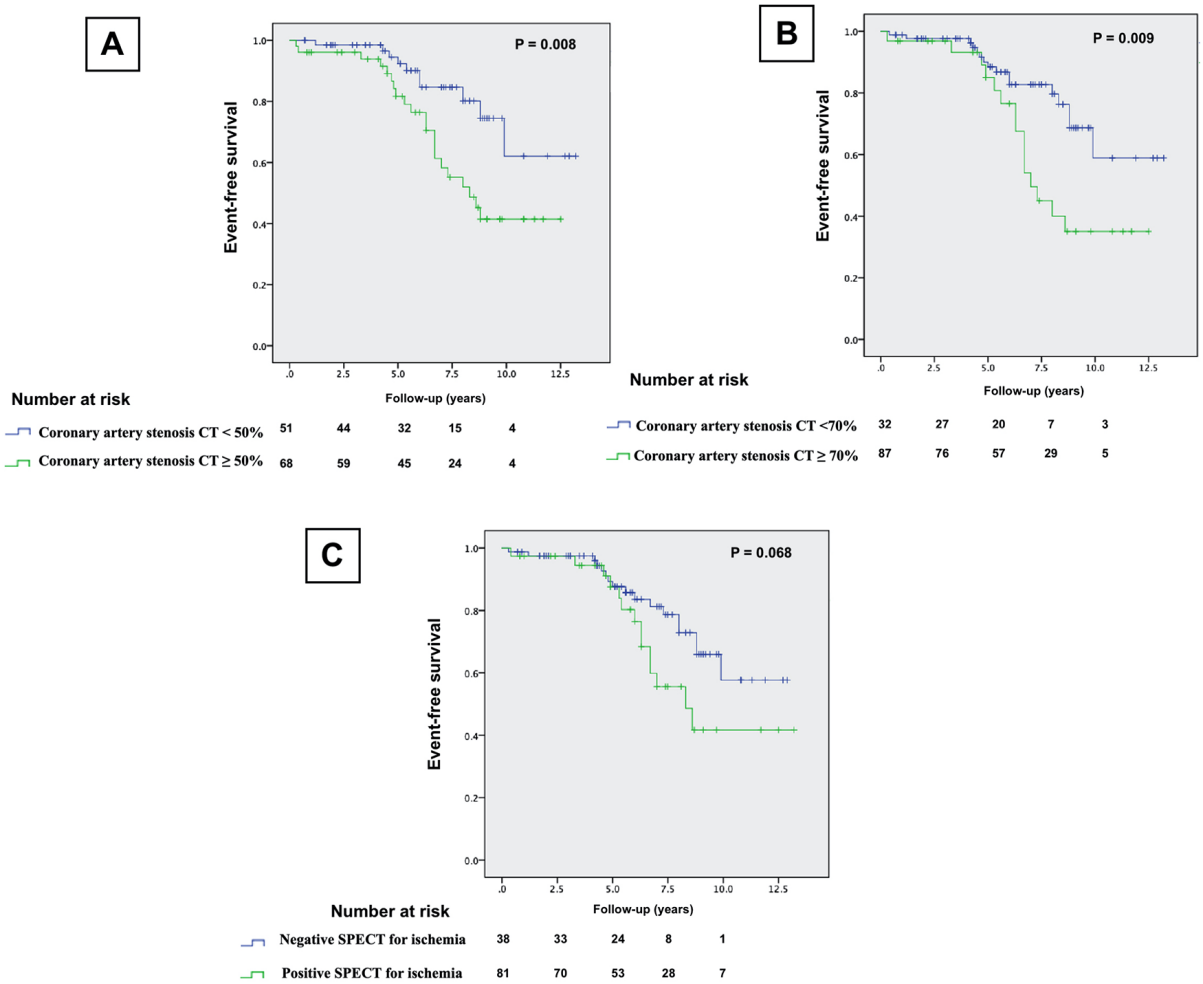
Kaplan-Meier HCE-free survival curves, (**A**): CCTA coronary stenosis ≥50% stenosis compared to <50% stenosis, (**B**): CCTA coronary stenosis of greater than 70% versus <70%, and (**C**): patients with and without revisable ischemia on SPECT.

Kaplan-Meier survival analysis showed that coronary stenosis of greater than 50% or 70% and presence of revisable ischemia with SPECT are significant predictors of total MACE (*p *= 0.016, 0.033 and 0.023), respectively, Figure 2. However, CT stenosis were significant predictors of HCE (*p* = 0.008 and 0.009), while abnormal MPI was not a predictor of HCE (*p *= 0.068), Figure 3.

## Discussion

To our knowledge, this is the first study to evaluate and compare the prognostic value of hybrid CCTA / SPECT in patients with CAD treated with PCI with a reasonable follow-up period. We found that critical stenosis on CCTA can predicts both MACE and HCE while abnormal SPECT can only predict MACE but not HCE.

CCTA is an important imaging modality for the diagnosis of coronary stenosis. It is well-validated in the prediction of cardiovascular events in patients with suspected CAD and post-revascularization with coronary artery bypass graft surgery (11–13). However, the predictive value of CCTA post-PCI is limited and yet to be established (14).

Hybrid imaging is increasingly being used in the diagnosis and the prediction of long-term outcome in CAD (9). Anatomical details of the coronary artery stenosis gathered from CTA or ICA such as: severity, number of lesions and location of the affected coronary segments are integral to identify population at higher risk for cardiovascular events (15, 16). Similarly, myocardial perfusion defects data driven from functional imaging such as number, severity and the extent can predict MACE and long-term outcomes (17). Combined anatomical and physiological imaging has an advantage through compensating for the weak properties of each procedure and dissolving the nonconclusive results of each stand-alone imaging modalities (18).

Chen MY, et al. found that combined CCTA and CT perfusion has similar prediction of MACE, compared to that of ICAG and SPECT (9). Rispler showed that Hybrid CCTA/SPECT resulted in improvement of the specificity and the positive predictive value in the detection of hemodynamically significant coronary stenosis in individuals with suspected CAD (5). In contrast, Danad I et al., demonstrated that the combination of functional and anatomical imaging does not add diagnostic value but can guide the clinical decision-making in patients with CAD (19).

In our study, CCTA was a significant predictor of MACE and HCE similar to Hossain (14) who found that coronary stenosis of >50% on CCTA had significant prognostic value and can independently predict MACE in patients with coronary stents.

Accuracy of stent interpretation is mainly affected by partial volume effects and blooming artifacts, more prominent with smaller stents, with only 13%—26% reported to be interpretable (11, 12). In our study, one-fourth of the stents were less than 3 mm and approximately 20% of all stents were non-interpretable.

Limitations of our study include being a single center study with a small number of patients. Since stent visualization is improving through hardware and software innovations, hybrid imaging is promising. Our results cannot be generalized to independent interpretation of CCTA and SPECT in patients with stents, since SPECT and CTA were interpreted together by physicians trained and experienced in both areas. Only 84% of the patients could be followed up and this may compromise the external validity of the study.

## Conclusions

CCTA measurements of coronary artery stenosis are significantly associated with MACE and HCE in patients with coronary stents. On the other hand, detection of reversible ischemia with SPECT has a significant association with MACE but not HCE.

## Data Availability

The raw data supporting the conclusions of this article will be made available by the authors, without undue reservation.
